# Editorial: *Setaria* as a Model Genetic System to Accelerate Yield Increases in Cereals, Forage Crops, and Bioenergy Grasses

**DOI:** 10.3389/fpls.2019.01211

**Published:** 2019-10-10

**Authors:** Andrew N. Doust, Thomas P. Brutnell, Hari Deo Upadhyaya, Joyce Van Eck

**Affiliations:** ^1^Oklahoma State University, Stillwater, OK, United States; ^2^Chinese Academy of Agricultural Sciences, Beijing, China; ^3^CGIAR Research Program on Climate Change, Agriculture and Food Security (CCAFS), South Asia, New Delhi, India; ^4^Boyce Thompson Institute, Ithaca, IL, United States

**Keywords:** *Setaria*, Green foxtail, foxtail millet, C4 photosynthesis, grass, Poaceae

*Setaria* is a model C4 grass in the tribe Paniceae of the subfamily Panicoideae, closely related to switchgrass, napier grass, and, pearl millet, and, in the sister tribe, closely related to important crops such as maize, sorghum, and sugar cane. The model comprises two species, foxtail millet (*Setaria italica*), domesticated in the Yellow River valley in China approximately 9–11,000 years ago, and its wild progenitor, green foxtail (*Setaria viridis*), which is one of the world’s most widespread weeds. *Setaria*, particularly green foxtail, differs from its more important biofuel and crop relatives in that it is small in stature and fast cycling and has a small genome. As such, it is ideally suited to greenhouse and growth experiments, as well as being capable of being grown in field plots. Over the last 10 years, numerous resources have been created for both species, including annotated genomes, mutant and field collections, transformation protocols, and gene atlases. Some of these advances were detailed in a volume in the Springer Crop Genetics and Genomics volume released in 2017 (Doust and Diao, 2017). The current collection of papers in this Research Topic, covering domestication, developmental genetics, mutant analysis, microbiomes, and technical advances, shows how far the field has progressed even in the short time since that volume.

Several papers deal with the genetics of domestication and improvement. Hu et al. present an overview of domestication and improvement in *Setaria*, focusing on key traits that differ between foxtail millet and its wild progenitor green foxtail. Concentrating on the domestication and improvement traits of shattering, plant architecture and flowering time, the paper summarizes known information and points out new opportunities for improvement in drought stress and nutrient efficiencies.

Odonkor et al. take the theme of domestication further and investigate the genetic basis of shattering in *Setaria* in more detail. Through mapping and gene expression analyses, the authors pinpoint the ortholog of sorghum SH1 as being likely to underlie the quantitative trait locus (QTL) of the largest effect, with a second small QTL in the vicinity of the ortholog of rice qSH1. These results are significant because they suggest that there might be a common genetic control of shattering across the grasses. Chaluvadi and Bennetzen also look at the effects of domestication but focus belowground, contrasting soil microbiome differences between wild and domesticated *Setaria*. They point out key differences in the microbiotic assemblages of domesticated versus wild accessions and suggest that domestication has selected for specific associations in the root and rhizosphere.

Other papers deal with gene expression analyses. Zhu et al. present a co-expression study of early inflorescence development in green foxtail, which is canonical with that found in other panicoid grasses even though the resulting inflorescence is more complex. Six developmental stages were identified, containing stage-specific co-expression modules with homologs of known developmental genes from maize and rice, suites of transcription factors, and unknown genes. This study will be important for comparative analyses and gene discovery in *Setaria*.

Three papers detail screening and characterization of mutants related to C4 photosynthesis, Kranz structure, and chloroplast biogenesis in foxtail millet. Luo et al. detail their studies on identifying ethyl methanesulfonate (EMS) mutants of foxtail millet that show variation in anatomical structure, especially related to Kranz anatomy. The genetic basis of Kranz anatomy, an essential part of C4 photosynthesis in grasses, has been exceedingly difficult to elucidate, and the 14 mutants identified by Luo et al. represent an important new set of resources for genetic analysis. Another two papers describe the identification of genes responsible for chloroplast biogenesis and a range of other phenotypes. Zhang, Tang, et al. identify the *Setaria* ortholog of deoxycytidine monophosphate deaminase (DCD), a key enzyme in dTTP biosynthesis. Surprisingly, the expression of this gene in *Setaria* differs from that in rice (a C3 plant) and is affected by low CO_2_ environments, showing similar patterns of expression to C4 photosynthesis genes. This may provide new avenues for understanding the differing roles of this essential gene in C3 and C4 grasses. Zhang, Zhi et al. describe the identification of an ATP-independent metalloprotease that is required for chloroplast development, photosystem II function, leaf senescence, and abscisic acid (ABA) signal response. This is the first report characterizing this gene’s functions in C4 plants.

*Setaria* is also an excellent vehicle for stress studies, including drought, salt, and low nutrient levels. The paper by Kaur et al. details the role of an *Arabidopsis* type III Gγ protein AGG3 in monocots, using the over-expression of the *Arabidopsis* gene in *Setaria* to gain insight into its function in C4 grasses. In doing so, Kaur et al. reveal that several traits are correlated with gene expression level while others appear to exhibit allele-specific expression. Traits related to stress were positively affected, including responses to salt and low nitrogen.

The papers that seek to understand the genetic control of development and response to the environment all rest on a requirement for accurate and sensitive phenotyping. Two papers directly address this issue. Acharya et al. conduct multiple phenotypic assays of *Setaria* to optimize growth conditions under multiple hormonal and abiotic stresses. This is very important to provide a secure base of information for the *Setaria* community to conduct further phenotypic assays. A very different tack is taken by Desai et al., who use neural net classification combined with movement analysis of time-lapse imagery to track flower opening in *Setaria*. This trait is particularly tricky to track in *Setaria*, where the inflorescence architecture is complex, and flowers open briefly and in different parts of the inflorescence. Knowledge of timing of anther appearance is critical for the success of controlled crosses in *Setaria* as well as in breeding for favorable opening times in hot field environments.

Finally, Van Eck provides a review of *S. viridis* transformation approaches. In it, she discusses tissue culture-based and floral dip approaches and concludes that tissue culture-based methods are more reliable, though more technically demanding, than the floral dip method. Simplification of tissue-based approaches and further optimization of the floral dip method—the holy grail of monocot transformation—are necessary for *Setaria* to reach its full potential as a model system.

While by no means being a comprehensive review of all activity in *Setaria* to date, these articles provide a unique insight into the value and potential of the *Setaria* model system.

## Author Contributions

AD wrote manuscript; JE, HU, and TB provided comments and edits.

## Conflict of Interest

The authors declare that the research was conducted in the absence of any commercial or financial relationships that could be construed as a potential conflict of interest.

## References

[B1] Doust A. N. Diao X. (eds.) (2017). The genetics and genomics of *Setaria* . Plant genetics and genomics: crops and models, vol. 19 Springer-Verlag, New York. 10.1007/978-3-319-45105-3

